# Creep Behavior of A356 Aluminum Alloy Reinforced with Multi-Walled Carbon Nanotubes by Stir Casting

**DOI:** 10.3390/ma15248959

**Published:** 2022-12-15

**Authors:** L. Shan, C. Y. Tan, X. Shen, S. Ramesh, R. Kolahchi, M. H. Hajmohammad, D. K. Rajak

**Affiliations:** 1 Department of Mechanical Engineering, Faculty of Engineering, Universiti Malaya, Kuala Lumpur 50603, Malaysia; 2 Centre of Advanced Manufacturing and Materials Processing (AMMP), Faculty of Engineering, University Malaya, Kuala Lumpur 50603, Malaysia; 3 State Key Laboratory of Mechanics and Control of Mechanical Structures, Nanjing University of Aeronautics and Astronautics, Nanjing 210016, China; 4 Huanghe Jiaotong University, Zhengzhou 454950, China; 5 School of Materials Science and Engineering, State Key Laboratory of Silicon Materials, Zhejiang University, Hangzhou 310027, China; 6 Department of Mechanical Engineering, Imam Hossein University, Tehran 1698715461, Iran; 7 Indian Institute of Technology (ISM), Dhanbad 826004, JH, India

**Keywords:** aluminum alloy, stir casting, nanocomposites, MWCNTs, creep

## Abstract

Lightweight aluminum alloy components are often used to manufacture a variety of engineering components in many industries. In recent years, researchers have studied the effect of improving the mechanical properties of metal alloys by incorporating nano-carbon into its structure. In this study, the effect of the addition of 0.2, 0.5, and 1 wt% of multi-walled carbon nanotubes (MWCNTs) on the stress–strain behavior and creep phenomenon of an A356 aluminum alloy were studied. The effect of nickel coating on 0.2 wt% MWCNTs was also investigated. Samples were prepared using the stir-casting method. The results revealed that the grain size became finer when MWCNT nano-particulates were introduced. Although the MWCNTs were distributed homogeneously in the A356 matrix, as confirmed by FESEM analysis, there were some agglomerations observed in a specific area with dimensions smaller than 100 nm. Nevertheless, the addition of MWCNTs was found to be beneficial in enhancing the hardness of alloys containing 0.2 wt%, 0.2 wt% nickel-coated, 0.5 wt%, and 1 wt% MWCNTs by 9%, 24%, 32%, and 15%, respectively, as compared with the unreinforced A345 matrix. It was also found that the 0.5 wt% MWCNT-A356 matrix exhibited an improvement in the creep lifetime by more than two orders of magnitude.

## 1. Introduction

Aluminum nanocomposites have been extensively used in applications such as aerospace, military, and automotive due to their remarkable physical and mechanical characteristics. Compared with conventional monolithic materials, aluminum nanocomposites exhibit high strength [1,2], excellent abrasion [3] and corrosion resistance [4,5,6], high fatigue strength [7], high creep resistance [8], a low thermal expansion coefficient [9], and a low weight to strength ratio [10]. Meanwhile, many studies have been performed in this field to enhance this alloy’s mechanical behavior. For example, Elhadari et al. [11] investigated the influence of doping certain elements in Al alloy on the fatigue behavior and tensile performance of simple and heat-treated samples. According to the results of their tensile experiments, the ultimate tensile resistance was enhanced by introducing Ti, Zr, and V nanoparticles. Many other studies on improving the properties of Al alloys have been carried out by other researchers. Heshmati et al. [12] studied the vibrational and mechanical properties of carbon-nanotube-reinforced composites. Based on the polyvinyl-chloride foam core and glass–fiber face sheet, Patra and Mitra [13] performed an experiment to investigate composite sandwich materials. Ajoku conducted an experiment involving two different composites of aluminum foam formed through the powder metallurgy process with recycled ballistic aggregates added in regular and random patterns [14]. The mechanical properties of this composite were examined under compression loading by using a universal testing machine and image correlation then compared to the properties of aluminum and steel foams produced in the laboratory and found in the literature, respectively. Ajoku found that the results for aluminum foam ceramic composite show an increase in its yield strength, plateau stress, relative density, and strength/density ratio, but a decrease in its Young’s modulus. Linul et al. [15] investigated the influence of thermal load on the mechanical properties of closed-cell tubes filled with aluminum-alloy foam and subjected to compressive quasi-static loads. The mechanical behavior of the closed-cell aluminum-alloy foams subjected to various quasi-static loads in thermal condition was presented by Linul et al. [16] as well. In another study, Prakash et al. [17] confirmed that producing the Al/CNT nanocomposites with the stir casting method can increase the homogeneity of nano-additive dispersion throughout the metal matrix, and the mechanical characteristics can thereby be improved. In another study, Lee et al. [18] employed the stir-casting technique to prepare the Al nanocomposite specimens and found that by increasing the B_4_C volume fraction up to 20%, the tensile resistance of B_4_C/Al-6061 was improved compared to the base Al alloy.

Divagar et al. [19] not only employed two kinds of nano additives to fortify the Al 7075 aluminum alloy matrix, but also used alumina at 5% by mass as well as SiC at 5%, 10%, and 15% by mass to fortify the base alloy by employing the stir-casting technique. Their results revealed that the addition of nano additives could enhance the fatigue behavior of the fabricated samples. Moreover, specimens with alumina at 5% by mass and silicon carbide at 10% by mass have the longest fatigue life. In addition, Raju et al. [20] examined the impact of changes in the percentage of alumina-reinforcing particles on the fatigue behavior of Al 2024. They used the ball-milling method to prepare the composite powder at 300 rpm for 2 h and preheated the reinforcing nanoparticles for 30 min at 700 °C. Furthermore, the nano-additives were immersed in the melt for 4 min at 200 rpm, and the specimens were cooled in an argon medium after tapping. They subjected the fabricated samples to fatigue and tensile tests. The experimental findings confirmed that the nanocomposite specimens had a higher tensile strength compared to the base alloy. Hemanth [21] used silica microparticles to reinforce the piston alloy. The matrix phase was mixed with silica with a particle size of 30–300 mm and volume fraction of 3–12%. The materials were melted at 700 °C, and the stir casting technique was employed to fabricate the nanocomposite. At the same time, they reported that with the addition of 9% of silica by mass, the yield strength and ultimate tensile resistance were both considerably improved. Mazahery et al. [22] utilized the stir casting technique to produce an Al_2_O_3_-reinforced Al cylinder head. In this work, the composites were prepared using ball-milling, and the mixture was melted at 800 °C prior to sample preparation. According to their findings, the addition of 2.5 wt% Al_2_O_3_ as the reinforcement phase could considerably improve the tensile properties of the Al cylinder head. As pointed out by Smart et al. [23], the addition of both TaC and Ti resulted in an increment in the resistance to deformation and the load capacity up to 500 kgf at the temperature of 120 °C in comparison to the base Al 7075 alloy. In terms of the creep characteristics, the test specimen with 0.5 wt% TaC, 6 wt% Si3N4, and 1 wt% Ti was able to resist more than 30,000 cycles at a strain rate is 0.02.

In recent studies, the mechanical properties of Al alloys were enhanced by adding selective amounts of nanoparticles and heat treatment and using different casting methods [24,25,26]. Rohatgi et al. [27] aimed to fortify the A206 Al alloy by employing two different kinds of nanoparticles, such as 5 wt% Mg and silica (5–9 wt%). In addition, they melted the materials at a temperature of 800 °C. After the tapping, the specimens were heat-treated. The authors found that the highest hardness was recorded for Al alloy containing Mg and 9 wt% silica. Ansari Yar et al. [28] employed MgO particles with a size of 50 nm to strengthen the A356.1 Al alloy using the stir casting technique. Their findings indicated that the reinforced specimens had higher hardness than the base specimen. Furthermore, the hardness increases as the melt temperature increases from 800 °C to 950 °C. Beyond that, the reinforced materials have improved tensile and compressive properties = compared to the unreinforced sample. Sajjadi et al. [29] performed an experimental examination to explore the influence of Al_2_O_3_ particle size (mm and nm) on the metallurgical characteristics and hardness of Al cylinder head by employing the stir-casting technique. According to their experimental results, using nano-sized Al_2_O_3_ led to a more uniform microstructure and higher hardness compared with specimens containing micron-sized alumina particles. Furthermore, they demonstrated that the wettability decreases with finer particle size [30] and the specimen containing 3 wt% alumina exhibited better compressive strength and hardness.

Zhao et al. [31] conducted ultrasonic-assisted compression tests to study the stress–strain characteristics of Al AA2024-T3 alloy under ultrasonic vibration. According to Bhowmik et al. [32], by adding both TiB2 and SiC nano additives to the Al 7075 alloy, the tensile strength and hardness improved by about 41.42% and 30.3%, respectively, compared with the unreinforced Al. Moreover, Panthglin et al. [33] emphasized that the addition of Zr with a weight percentage of 0.14% to A356-SiC nanocomposite could improve the creep behavior. However, their findings revealed that increasing the weight percentage of Zr higher than 0.14% caused deterioration in the creep strength. Xie et al. [34] investigated the mechanical performance of the Al matrix reinforced with graphene nano-platelets. In another study, Zhao et al. [35] examined the mechanical characteristics of Al-based nanocomposite under high-temperature conditions.

The use of some conventional monolithic materials such as Al and its alloys is very popular in industry due to the above-mentioned specific features [36,37]. However, in most cases, these materials have internal (microstructural) defects, which can be related to the production method (e.g., forging and die casting), environmental conditions (e.g., temperature and moisture), and type of application. One of the common techniques to repair or reduce these kinds of defects is to add nano-, micro-, and sub-micro-sized particles [38,39]. In this regard, many studies have been conducted, and they all mentioned the positive and adverse effects of adding particles to a material’s structure. In addition, Ajayan and Tour [40] and other researchers [41] suggested that better mechanical properties can be achieved by improving the microstructure of nano-additives such as carbon nanotubes. Other researchers [42] investigated the effects of adding SiC nanoparticles on the tensile properties of an Al 7075-T6 alloy and found that the SiC was beneficial in enhancing the tensile properties of the Al alloy. Hamedan and Shahmiri [43] reported the synthesis of A356 reinforced by SiC nanocomposite using a modified stir casting approach by studying the effects of the main parameters of this approach such as stirring temperature, stirring rate, and the main powder type on the mechanical properties and microstructure of nanocomposites. Their results indicate that with optimized parameters of modified stir casting (stirring temperature and stirring speed are at 750 °C and 700 rpm, respectively, and master powder reinforced by 20 wt% SiC nanoparticles), tensile tests demonstrated that with 1.0 wt% nanosized SiC, the yield strength and ultimate tensile strength of the nanocomposite improved, while the ductility is slightly decreased. Zeeshan et al. [44] studied the mechanical properties and microstructure of A356 alloy reinforced with 3 wt% of B_4_C (40 and 90 µm). The composite is prepared well using the stir method. They used ASTM scales to evaluate UTS, hardness, and yield quality. Mechanical properties such as hardness, UTS, and yield quality were evaluated by ASTM scales. They found that yield stress, UTS, and hardness of the composite were increased because of the essence of B_4_C particles in the composite.

Although there are some studies on the effects of nanocarbons as a reinforcement phase on the mechanical properties of various light alloys, however, there are no published data on the A356 aluminum alloy reinforced with multi-walled carbon nanotubes (MWCNTs). The main purpose of the present work was to investigate the mechanical properties and creep behavior of the Al-MWCNT nanocomposite.

## 2. Materials and Methods

The MWCNTs used in this work (purchased from Jiangsu XFNANO Materials Tech Co., Ltd., Nanjing, China), with an outer diameter of 20–30 nm and a length of 10–30 μm, were produced by the chemical vapor deposition process. The composition of the MWCNTs is given in Table 1.

A356 aluminum alloy (purchased from WUXI SUHECHENG Aluminum Co., Ltd., Wuxi, China) was used as the base alloy. Table 2 presents the elements percent in the A356 aluminum alloy. It was found that the composition conforms to ASTM Standard Specification B26/B26M-09 [45].

**Figure 1:**
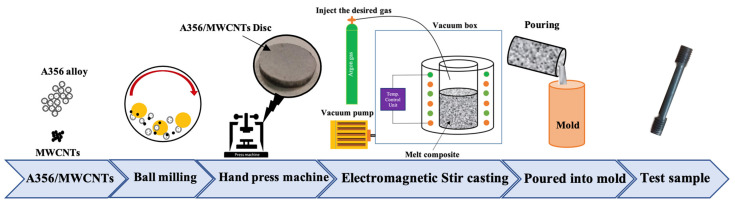
Schematic of manufacturing steps.

A schematic of the mixing and stir-casting method is shown in Figure 1. Since MWCNTs are exposed to temperatures lower than the melting point of aluminum (for example, temperatures below 650 °C), they are highly likely to be oxidized. Therefore, the melting operation must be performed in a vacuum. For this purpose, the presence of a chamber and a vacuum pump are necessary for casting. As for the stir casting method used in this work to produce samples, it could inject the desired gas into the melt during the melting process; however, it is possible that a vacuum environment will be created in the chamber. This furnace does not need a mechanical stirrer to stir the melt and the reinforcement. The stirring in this furnace was carried out using an electromagnetic stirrer with a frequency of 10 Hz. Apart from that, the ball-milling method was used to mix the MWCNTs under the condition of a mixing time of 1 h and a milling speed of about 200 rpm. Furthermore, argon gas was purged into the ball mill during mixing to prevent particle agglomeration [1]. This powder must enter the composite and melting stage inside the crucible and next to the ingot, which must be accomplished in vacuum conditions according to the temperature conditions of MWCNTs. It is noteworthy that creating vacuum conditions with the presence of powder causes the powder to be sucked by the vacuum pump. Therefore, the mixture of aluminum and MWCNTs powder was made in the form of discs using a hand press machine.

After the reinforcing particles of MWCNTs and aluminum ingots were prepared at suitable dimensions (60 mm diameter and 70 mm height), aluminum ingots and discs needed to contain the appropriate amount of MWCNTs particles and 1 wt% magnesium to increase the wettability of the MWCNTs in the Al matrix. In addition, the matrix phase was placed in a chamotte casting crucible and mixed at 100 rpm for 2 min [1]. Finally, the prepared melt was poured into the mold. By repeating the same steps, nanocomposites containing nanoparticles with weight percentages of 0.2%, 0.5%, and 1% were made.

**Figure 2:**
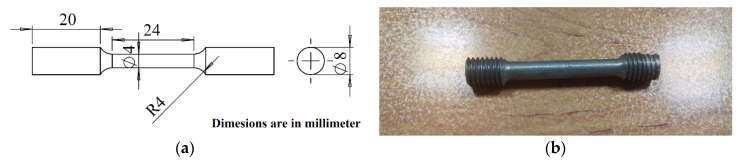
Specimen for the tensile test based on ASTM-E8: (**a**) 2D diagram; (**b**) fabricated specimen.

At the same time, the molded cylinders were machined and fabricated based on ASTM-E8 for tensile testing. In order to examine the effect of coated MWCNTs on mechanical performance, a nanocomposite with a weight percentage of 0.2% was also coated with nickel (i.e., 0.2% Ni-Coated MWCNTs) using the electroless polymer-nickel coating method. After the multi-wall carbon nanotubes were coated with nickel ferrite particles, the powder with a mean particle size of 20–30 nm, taken from the filter, was placed in the mechanical ball mill along with aluminum powder after drying in the furnace. After the disc was made according to the composite manufacturing process from MWCNTs, the samples of aluminum reinforced by MWCNTs/nickel (MWCNTs coated by nickel ferrite particles) were fabricated. Figure 2 shows a standard tensile test specimen. The tensile tests were carried out using the TSE-D device, (Wance, Series105D, Shenzhen, China) at room temperature and with a strain rate of 5 × 10^−4^ 1/s. The hardness of the samples was determined using the Brinel hardness method at a load of 62.5 kgf based on ASTM E10-15a: 2017 standard, and the microstructure of the base alloy and nanocomposites samples were investigated using FESEM (Hitachi, S-4800, Tokyo, Japan) with operating between 100 volts and 2 kV, in order to check the agglomeration of MWCNTs after stir casting and check integrity of MWNCTs dispersion in the A356 matrix. Moreover, the optical microstructure was used to observe MWCNTs in the matrix phase. An important goal in arming the A356 aluminum alloy with nano additives was to increase their efficiency for high-temperature applications. At the same time, the main part of the current research was to produce nanocomposites with suitable creep properties compared with the base A356 aluminum alloy. For this reason, in this study, specimens were fabricated and creep tests were performed based on ASTM-E8 standard. The experiments were performed using TSC-B device (Wance, 304B) under 300 °C and 50 MPa.

## 3. Results and Discussion

**Figure 3:**
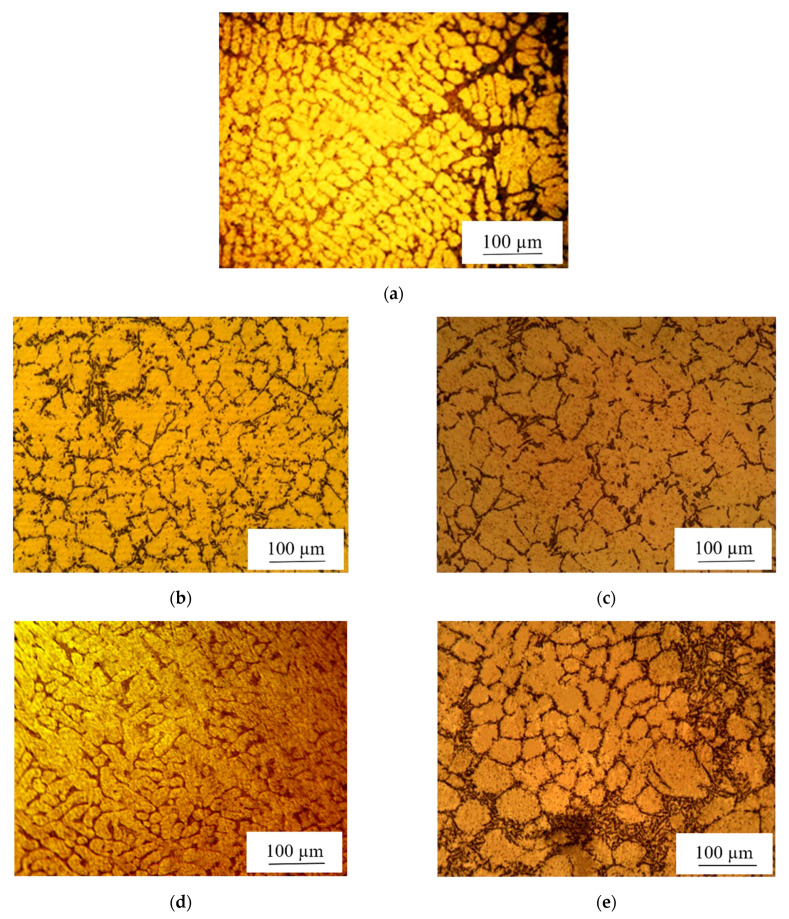
Typical optical images of (**a**) A356 alloy; Al alloy containing (**b**) 0.2% MWCNTs, (**c**) 0.2% coated-MWCNTs, (**d**) 0.5% MWCNTs, and (**e**) 1% MWCNTs.

The optical images of the A356 aluminum alloy and Al-nanocomposites are shown in Figure 3. When nanoparticles were added to the base alloy, the grain size decreased, as reported earlier by Hamedan and Shahmiri [43]. According to the research by Bradbury et al. [46], with the decrease in grain size, the specimen’s hardness was improved. Isa et al. [47] reported that the reason behind the size reduction was the localized limitation of grain growth by the surrounded particles.

**Figure 4:**
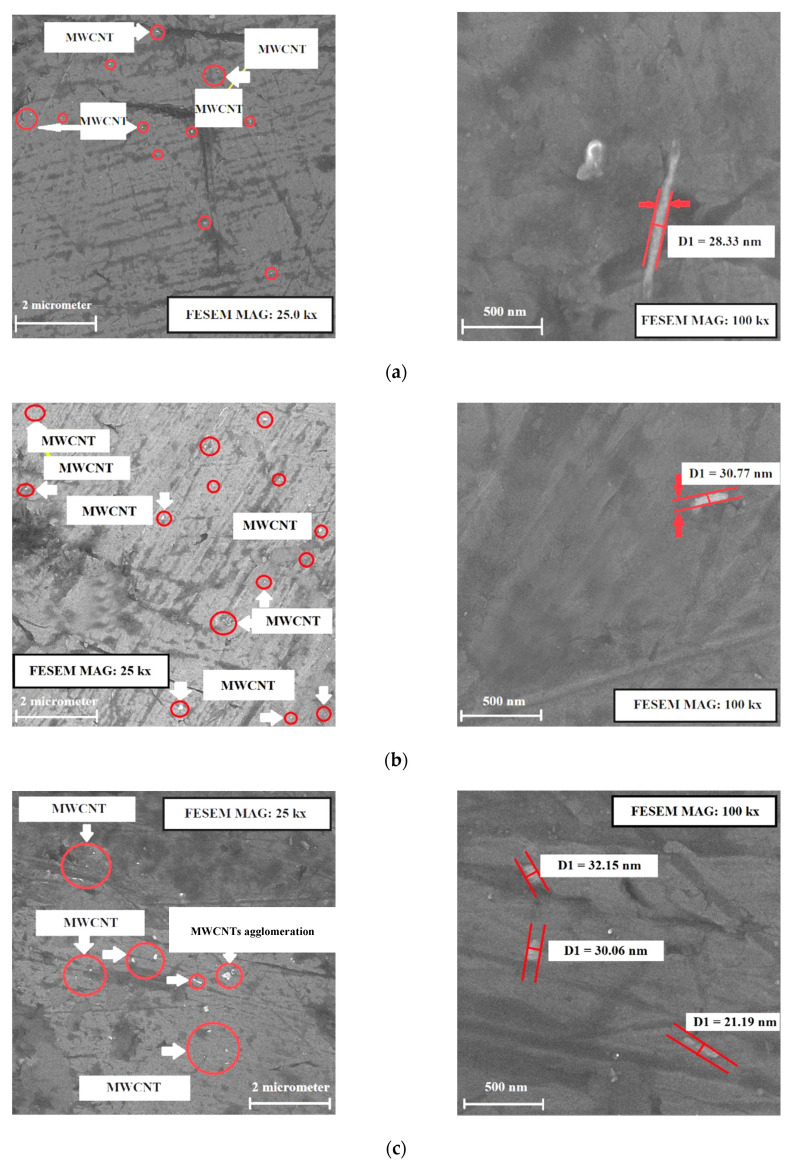

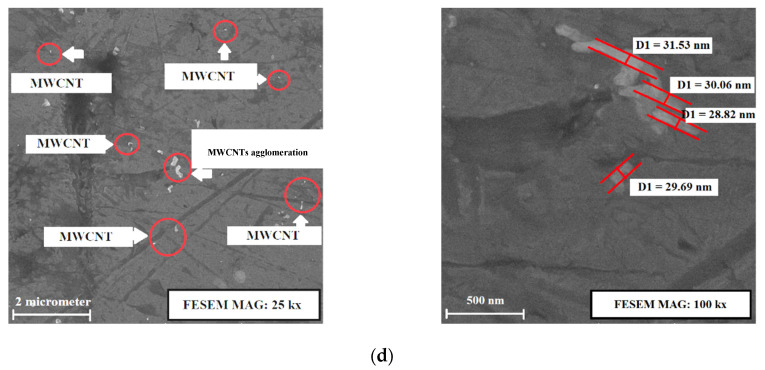
FESEM pictures of (**a**) Al 0.2% MWCNTs; (**b**) Al 0.2% coated-MWCNTs; (**c**) Al 0.5% MWCNTs; (**d**) Al 1% MWCNTs–FESEM images.

FESEM images were used to evaluate the fabrication quality of nanocomposites. One of the issues discussed in making nanocomposites by casting method is to study the distribution of reinforcing particles throughout the matrix. Furthermore, the uniform particle distribution and the lack of particle aggregation zones will improve the mechanical performance of the nanocomposite in comparison to the base alloy. In addition, the existence of a strong bond between the arming MWCNTs and the Al alloy matrix was a factor increasing the strength of the specimen [20,48]. Meanwhile, the absence of porosity was another important parameter in casting [48]. In terms of the specimen production inspection, from a metallurgical perspective, Figure 4 shows the FESEM images of MWCNTs dispersed in the A356 aluminum alloy. It can be seen that the size of MWCNTs in the sample of Al + 0.2% MWCNTs is lower than in other cases. In addition, in the case of MWCNT-coated Al + 0.2%, the size of nanoparticles is larger than Al + 0.2% MWCNTs since it has a coated layer in this case.

**Figure 5:**
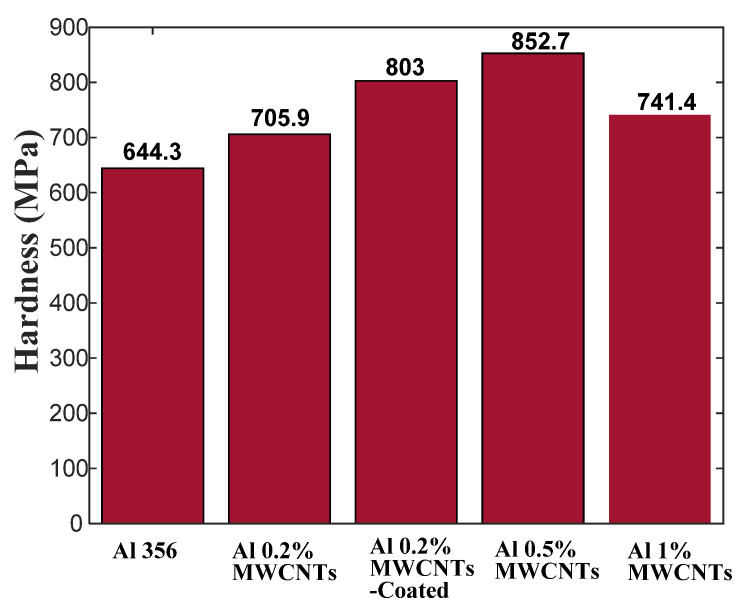
Brinell hardness of base alloy and nanocomposites made with different MWCNTs percentages.

As displayed in Figure 4, the dark background represents the A356 aluminum alloy, whereas the MWCNTs are represented by the lighter phase particles. Furthermore, the dispersion of nanoparticles in the Al alloy matrix is uniform, and agglomeration points are located in most areas with dimensions less than 100 nm. In general, according to [1], the specimens have the desired quality from a metallurgical perspective. From Figure 4a,b, it can be observed that the coated MWCNTs are better dispersed in the Al alloy matrix. Figure 4c,d show a rise in the number of MWCNTs, but the remarkable point is the cumulative distribution of raw particles of MWCNTs (especially in the case of a mass percentage of 1%). Hence, nanotubes tend to accumulate into clusters and bundles in the matrix, so dispersing them is the most challenging aspect. The hardness of the Al alloys is shown in Figure 5. The results show that the addition of multi-wall carbon nanotubes increases the hardness of the 0.2%, 0.2%-coated, 0.5%, and 1% Al specimens by 9%, 24%, 32%, and 15%, respectively, compared with the base alloy. The results obtained by Fullman et al. [49] revealed that the rapid increase in hardness at relatively low strains can be attributed to the formation of sub-grain boundaries and dislocation in the A356 aluminum alloy microstructure. Ansary Yar et al. reported that A356 aluminum alloy reinforced with 2.5 vol.% nano-sized MgO exhibited an increase of up to 56% in the hardness due to the presence of MgO particles with high hardness of 700 BHN [28]. Therefore, the hardness of the added nanoparticles affects the composite specimen significantly.

Figure 4d shows that the nanotubes tend to cluster in the Al matrix. The interaction between the matrix and the nanotubes is typically weak due to the special surface of nanotubes, which is atomically smooth. One of the serious problems of the composite lies in the fact that the nanotubes are difficult to disperse evenly in the matrix. When nanotube agglomerates in the composites are excessively stressed, they can slide along each other. This can cause cracks in the host layers and thereby weaken the composite. In fact, non-covalent interaction is a method of chemically modifying nanotube surfaces, which could be utilized to apply surfactants to the surrounding nanotubes or to adsorb the structures of aromatics on the outer walls [40]. The coating of the MWCNTs with nickel ferrite, as in the present work, proved to be beneficial in strengthening the Al matrix, as demonstrated by the higher hardness obtained for this sample in Figure 5.

**Figure 6:**
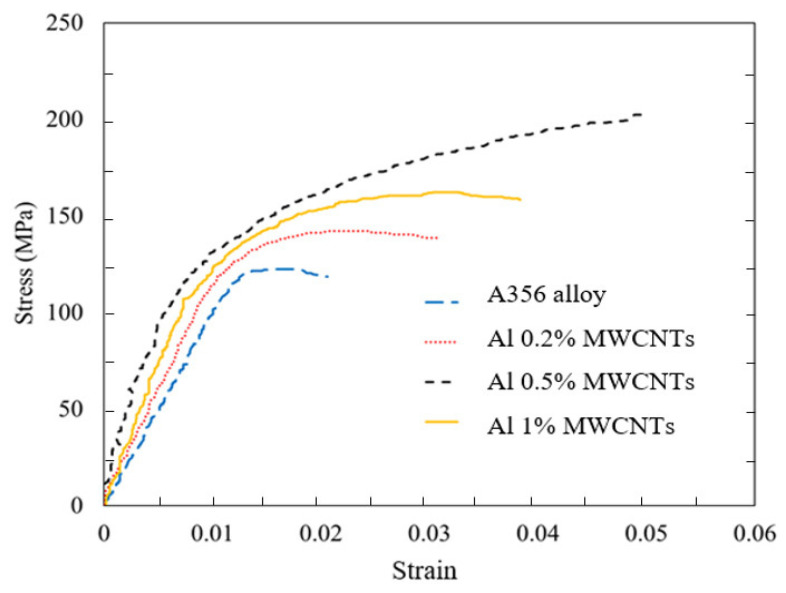
The stress–strain diagram of A356 aluminum alloy and nanocomposites containing 0.2%, 0.5%, and 1% MWCNT addition.

Figure 6 shows the stress–strain diagram of A356 aluminum alloy and nanocomposites made with 0.2%, 0.5%, and 1% MWCNTs, respectively.

**Figure 7:**
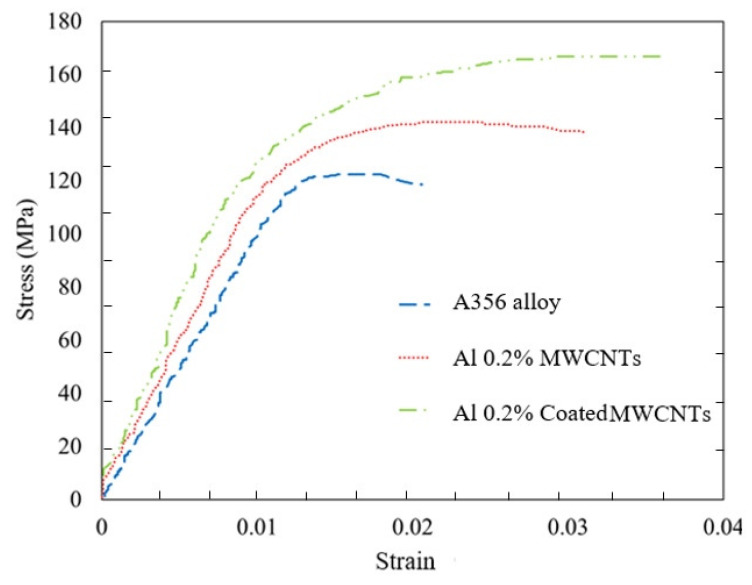
Stress–strain diagram of A356 aluminum alloy and 0.2% by mass nanocomposites with and without a coating of reinforcing particles.

Figure 6 reveals that, as the weight percentage of the MWCNTs increased from 0% to 0.5%, an increase was found in all the basic properties, such as yield stress, ultimate stress, elastic modulus, and fracture strain, as well as the areas under the stress and strain diagram. The stress–strain diagram of A356 aluminum alloy and 0.2% by mass nanocomposites with and without coating is shown in Figure 7. A more significant improvement in mechanical properties of nanocomposites with coated nanoparticles than that of uncoated nanoparticles.

**Figure 8:**
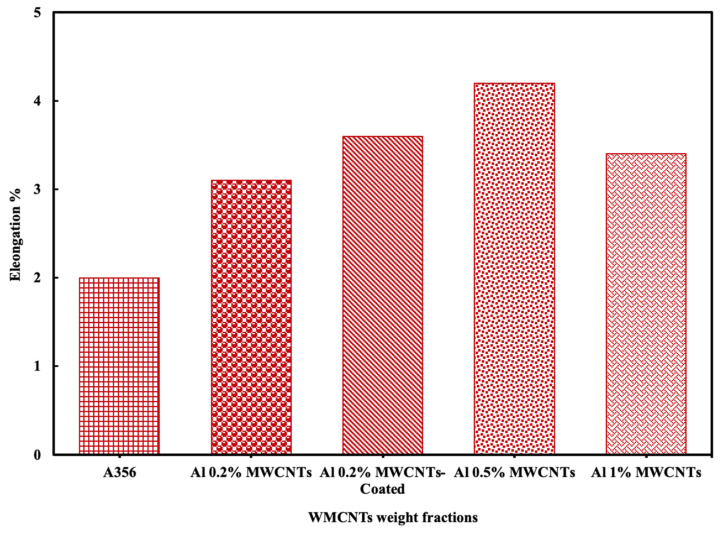
Effect of MWCNTs with different percentages on the elongation percentage of the A356 composite.

Table 3 shows the yield stress, ultimate tensile, hardness, and elongation at the break. On that basis, the yield stress and ultimate strength of the fabricated Al-MWCNTs compared with the base alloy increased. In terms of the ductility, Figure 8 shows that adding MWCNTs improves the composite ductility. The composite reinforced with 0.5 wt% MWCNTs showed a maximum elongation percentage of 4.2% compared to 2.0% for a monolithic alloy. Due to the interaction between MWCNTs and the alloy, the ductility and tensile strength of the composite simultaneously increased. This is ascribed to the slip-mode transition produced by the MWCNTs [50] due to the fact that MWCNTs are very small precipitates, the plastic deformation switches from dislocation reinforcement shearing to bypassing. The high strength of MWCNTs, however, impedes the dislocation motion and collapses them around the MWCNTs/alloy interface. [51]. Ductility is increased due to the activation of these cross-slip modes. During this study, similar results were obtained to those obtained by other researchers working with aluminum magnesium alloys. [52,53]. The mechanism responsible for the simultaneous increase in ductility will be further investigated in the future.

As shown in Figure 4, the addition of reinforcing particles modified the microstructure. At the same time, as the grain size becomes finer, and it is anticipated that the tensile properties improve [54]. Compared to A356 aluminum alloy, the tensile strength of reinforced specimens also increased. The highest increase was associated with the specimen reinforced with 0.5% MWCNTs. The nanocomposite with 0.5% MWCNTs has the highest yield stress, maximum hardness, and highest elongation percentage compared to other specimens. However, the addition of reinforcing particles generally increased the hardness, ultimate tensile resistance, and elongation percentage compared with the A356 aluminum alloy, and their percentage of increase compared with the base alloy is presented in Table 4. These results show that the mechanical properties of MWCNT-reinforced A356 aluminum alloy were higher than those of ZrO_2−_ and SiC-reinforced A356 aluminum alloy, as reported in the literature [55,56].

**Figure 9:**
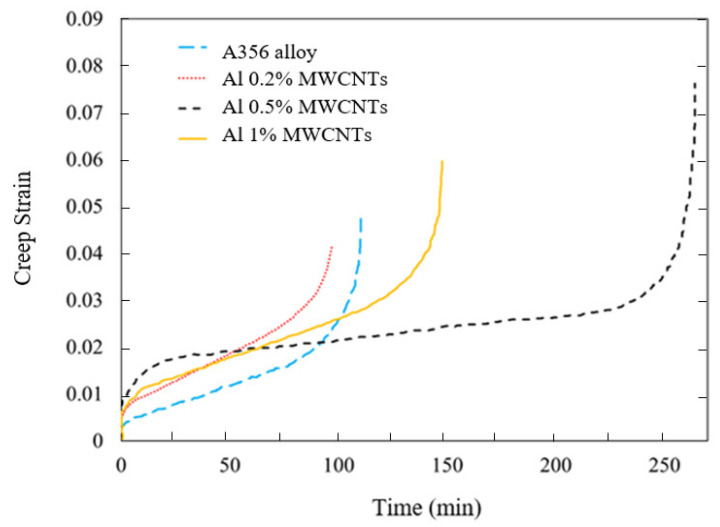
Creep strain diagram in time for A356 aluminum alloy and uncoated nanocomposites made with 0.2%, 0.5%, and 1% MWCNTs.

**Figure 10:**
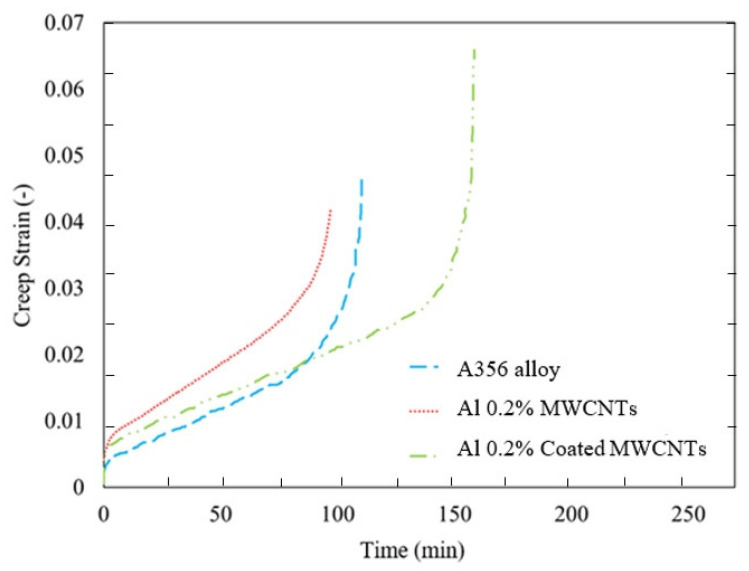
Creep strain diagram in time for A356 aluminum alloy and nanocomposites made with 0.2 and 0.2% Ni-coated MWCNTs.

The results of the creep test in the form of a creep strain diagram in terms of time are shown in Figure 9 and Figure 10 for the load 520 N. In addition, Table 5 presents the creep lifetime of A356 aluminum alloy and nanocomposites containing 0.2% MWCNTs, 0.2% Ni-coated MWCNTs, 0.5% MWCNTs, and 1% MWCNTs. It can be observed from Table 5 that the addition of MWCNTs with weight percentages of 0.5% and 1% increases the creep lifetime by 139% and 34%, respectively, but the addition of MWCNTs with 0.2% by weight reduces the creep lifetime by 12%. Apart from that, Al 0.5% MWCNTs show the optimum creep lifetime, and the incremental weight percentage of MWCNTs plays an insignificant role in increasing the creeping lifetime. However, coating the MWCNTs and adding it to the base alloy with the same weight percentage increases the creep lifetime by 43% compared with the A356 aluminum alloy. It is noteworthy that the first creep region for the base alloy and the nanocomposites is short and similar to the other, and the second regions of the base alloy and the nanocomposites are almost identical with 0.2% and 1%. However, the nanocomposite with a reinforcement of 0.5% by weight has a larger second area.

Table 5 shows the creep strain and fracture strain of the base alloy and the nanocomposites. It can be seen that the addition of MWCNTs with weight percentages of 0.5% and 1% increases the creep fracture strain by 62 and 30% compared with A356 aluminum alloy. Otherwise, the aluminum matrix with 0.2% MWCNTs reduces the creep failure strain by 13%. However, by coating the MWCNTs and adding them to the base alloy with the same weight percentage, the creep failure strain increases by 40% compared with the base alloy.

**Figure 11:**
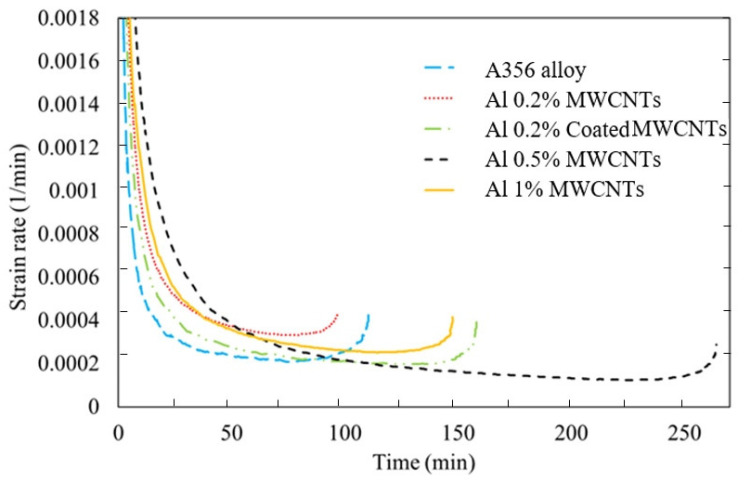
Creep strain rate–time diagram for A356 aluminum alloy and nanocomposites made with 0.2%, 0.2%-coated, 0.5%, and 1% MWCNTs.

Figure 11 is a diagram of the minimum creep strain rate over time, and Table 6 shows the minimum creep strain rate of the base alloy and nanocomposites. It can be seen from Table 6 that the minimum creep strain rates of nanocomposites made with 0.2% MWCNTs, 0.2% Ni-Coated MWCNTs, 0.5% MWCNTs, and 1% MWCNTs compared with A356 aluminum alloy have a 0.46% increase, 2.8% decrease, 32% decrease, and 3.2% decrease, respectively.

Overall, this study indicated that the addition of MWCNTs by 0.5% enhances the creep life, and Ni-coated MWCNTs show a positive effect on the creep behavior of the fabricated nanocomposite.

## 4. Conclusions

This paper studied the effect of adding MWCNTs on the mechanical performance and creep behavior of A356 aluminum alloy. The MWCNTs were added to the A356 matrix in weight percentages of 0.2%, 0.5%, and 1%. Additionally, MWCNTs with a mass percentage of 0.2% were coated with nickel ferrite to investigate the impact of the coating factor on the reinforcing particles. The results indicate that the addition of MWCNTs could enhance mechanical properties such as creep life, yield stress, ultimate tensile, and hardness. More specifically:

- The addition of MWCNTs enhances the hardness, and the maximum value achieved with 0.5% MWCNTs was 87 BHN, showing a 32% increase in comparison to the base alloy.
- When increasing the weight percentage from 0% to 0.5%, all the basic factors of mechanical performance such as ultimate stress, yield stress, elastic modulus, fracture strain, and toughness increase.
- The improvement in the mechanical and creep properties of nanocomposites with coated nanoparticles was more significant than that of uncoated nanoparticles. Furthermore, the yield stress increased by 37%, the ultimate tensile stress by 20%, the maximum hardness by 14%, and the maximum elongation by 16% in comparison to the uncoated nanoparticles.
- The nanocomposite with 0.5% MWCNTs had the highest yield stress, ultimate tensile stress, hardness, and elongation percentage compared with other specimens. In addition, the yield stress increased by 110%, the maximum ultimate tensile stress by 66%, the maximum hardness by 32%, and the maximum elongation by 110% compared with the base alloy.
- The addition of MWCNTs with weight percentages of 0.5% and 1% improves the creep life by 139% and 34%, respectively. However, the addition of MWCNTs with 0.2% weight percentages reduced the creep life by 12%.
- The addition of MWCNTs with weight percentages of 0.5% and 1% significantly enhances the creep failure strain by 62% and 30% in comparison to the A356 aluminum alloy.

## Figures and Tables

**Table 1:** MWCNTs’ chemical mixture.

C%	Al%	Cl%	Co%	S%
97.46	0.19	1.02	1.09	0.24

**Table 2:** Percentage of A356 aluminum alloy elements.

Al	Si	Mg	Fe	Cu	Mn	Ti
Base	7.03%	0.37%	0.15%	0.01%	0.02%	0.13%
ASTM standard	6.5–7.5%	0.25–0.45%	<0.2%	<0.2%	<0.1%	<0.2%

**Table 3:** Mechanical characteristics of the A356 aluminum alloy and fabricated nanocomposites.

Alloy	Yield Stress (MPa)	Ultimate Tensile Strength (MPa)	Hardness (MPa)	Elongation at Break (%)
A356	61	124	644 ± 66	2.0
Al 0.2% MWCNTs	82	148	705.9 ± 72	3.1
Al 0.2% MWCNT-Coated	112	178	803 ± 82	3.6
Al 0.5% MWCNTs	128	206	852.7 ± 87	4.2
Al 1% MWCNTs	90	159	741.4 ± 76	3.4

**Table 4:** Percentage increase in yield stress, tensile resistance, hardness, and elongation of fabricated nanocomposites compared with A356 aluminum alloy.

Alloys Compared with the Base Alloy	Yield Stress (%)	Ultimate Tensile Strength (%)	Hardness (%)	Elongation at Break (%)
Al 0.2% MWCNTs	34	19	9	55
Al 0.2% MWCNT-Coated	84	44	24	80
Al 0.5% MWCNTs	110	66	32	110
Al 1% MWCNTs	48	28	15	70

**Table 5:** Creep strain failure and lifetime of A356 aluminum alloy, nanocomposites with 0.2%, 0.2%-coated, 0.5%, and 1% MWCNTs.

Alloy	A356	Al 0.2% MWCNTs	Al 0.2% MWCNT-Coated	Al 0.5% MWCNTs	Al 1% MWCNTs
Creep Failure Strain (mm/mm)	0.0473	0.0417	0.0695	0.0762	0.0610
Creep lifetime (min)	110.4	97	158.4	264	147.4

**Table 6:** Minimum creep strain rate of A356 aluminum alloy and nanocomposites made with 0.2%, 0.2%-coated, 0.5%, and 1% MWCNTs.

Alloy	A356	Al 0.2% MWCNTs	Al 0.2% Coated-MWCNTs	Al 0.5% MWCNTs	Al 1% MWCNTs
Minimum Creep Strain Rate (min^−1^)	0.000428	0.000430	0.000416	0.000288	0.000414

## Data Availability

The data presented in this study are available from the corresponding authors upon reasonable request.
